# Supplementary material for: Post-lockdown SARS-CoV-2 nucleic acid screening in nearly ten million residents of Wuhan, China
Source: Nat Commun. 2020 Nov 20;11:5917. doi: 10.1038/s41467-020-19802-w (PMC7679396; doi:10.1038/s41467-020-19802-w)
Supplement: Supplementary file 1 — Supplementary Information [file 41467_2020_19802_MOESM1_ESM.pdf]

Supplementaries

Supplementary note 1. Testing protocol, assay methods, genes used and other criteria

Supplementary note 2. Diagnosis and definition of confirmed cases with COVID-19

Supplementary Table 1. Median age-stratified Ct-value of the samples of asymptomatic cases

Supplementary note 1. The testing protocol, including the assay method used, the gene used for detection or other criteria

Notes to S1: This is a English summary of key information from two Chinese official guidelines:

- Guidance on Standardized Quality Control of Laboratory Testing of COVID-19 in Asymptomatic Cases (in Chinese)
- National Guidance on Laboratory Testing Technology for COVID-19 (in Chinese)

Collection of specimens

All specimens were oropharyngeal swab samples. The sampling process strictly followed the disinfection procedure to ensure environmental ventilation and prevent cross-infection. Technicians who worked in COVID-19 specimen collection have been trained with biosafety and equipped with relevant experimental skills. Personal protective equipment requirements for sampling personnel: N95 and above respirators, goggles, one-piece protective clothing, double latex gloves, and waterproof boot covers. The specimens of the hospitalized cases were collected by the medical staff of the hospital. The specimens of close contacts were collected by local designated disease prevention and control agencies or medical institutions.

Package of specimens

All collected specimens were stored in a refrigeration-resistant sample collection tube with a spiral cover and a gasket inside. Sealed samples were further packed in a sealed bag, each bag containing only one specimen.

Preservation of specimens

Specimens used for virus isolation and nucleic acid testing were tested as soon as possible. Specimens can be tested within 24 hours were stored at 4℃. Specimens that cannot be tested within 24 hours were stored at -70℃ or below. A special bank or counter was set up to keep specimens separately.

For long-distance transportation, dry ice and other refrigeration methods were used for preservation. Repeated freezing and thawing were avoided during specimen transportation.

Management of specimens and strains

Collected specimens and virus strains were managed by specially-assigned person, and the source, type and number of the virus strains and samples had been accurately recorded. Effective measures were taken to ensure the safety of the virus strains and samples, and to prevent from such incidents as misuse, malicious use, theft, robbery, loss and disclosure.

Examination of specimens

Standard operating procedures such as preparation of reagents, virus inactivation, nucleic acid extraction, amplification and detection, result analysis and reporting were compiled in accordance with the actual instructions, and were strictly followed in the testing procedure.

Virus inactivation Virus inactivation of oropharyngeal swab specimens was followed by nucleic acid extraction. Preheat the water bath box to 56 ℃ in advance. Remove the specimens that need to be inactivated from the sealed bag and put them into the test-tube shelf of the water bath pot. Put heavy objects on the specimen cover to prevent the collection tube from floating. Shake the specimen well every 10 minutes and inactivate it for 30-45 minutes. The inactivation temperature can be adjusted according to the temperature used to extract the reagent, but not lower than 56℃.

Nucleic acid extraction and sampling Nucleic acid extraction and sampling were performed in the biosafety cabinet. The automatic nucleic acid extraction method based on magnetic bead adsorption was adopted to ensure the safety of personnel and purity and efficiency of nucleic acid extraction.

The gene used for detection

Two target genes, including open reading frame 1ab (ORF1ab) and nucleocapsid protein (N),were simultaneously amplified and tested during the real-time RT-PCR assay.

Target 1 (ORF1ab):

Forward primer (F): CCCTGTGGGTTTTACACTTAA

Reverse primer (R): ACGATTGTGCATCAGCTGA

The probe (P): 5′-VIC CCGTCTGCGGTATGTGGAAAGGTTATGG-BHQ1-3’

Target 2 (N):

Forward primer (F): GGGGAACTTCTCCTGCTAGAAT

Reverse primer (R): CAGACATTTTGCTCTCAAGCTG

The probe (P): 5’-FAM-TGCTGCTGCTTGACAGATT-TAMRA-3’

The quality control

Indoor quality control For each batch of indoor quality control, one weak positive and three negative quality control samples (1 normal saline sample and 2 negative samples) were set up, and the three negative quality control samples were randomly placed in the middle of the clinical samples, which were involved in the whole process of testing together with the samples to be tested. Weak positive quality control shall be positive, and all three negative quality control shall be negative, and shall be considered under control. Otherwise, it is out of control and cannot issue a report. The cause should be analyzed in time and the sample should be re-tested if necessary.

Laboratory internal comparison Two or more test systems (instruments/reagents) should be used in the laboratory to compare the data to show the consistency of the test results. If the test procedure is manually operated, a comparison should be made between different operators.

Judgment of testing results

According to The Prevention and Control Plan of COVID-19 (fifth edition), a cycle threshold value (Ct-value) less than 37 was defined as a positive test result, and no Ct-value or a Ct-value of 40 or more was defined as a negative test result, while Ct-value between 37 and 40 suggested that the sample should be retested, if the retest result was still less than 40 and amplification curve has obvious peak, the sample can be determined as positive, otherwise, negative result would be reported.

Since there are some commercial testing institutions participated in this citywide nucleic acid testing, different testing reagents and testing methods were used in the specific testing process, the Ct value thresholds to judge the positive testing result were varied.

Confirming of cases

Laboratory confirmation of positive cases requires one of the following two conditions: (1) The detection results of COVID-19 2 targets (ORF1ab, N) in the same specimen were both positive by real-time fluorescence RT-PCR. If there is a positive test result for a single target, it needs to be re-sampled and re-tested. If it is still positive for a single target, it is determined to be positive. (2) It can

be judged as positive if a single target is positive in both specimens by real-time fluorescent RT-PCR, or if a single target is positive in both two samples of the same type.

Serum antibody detection

The colloidal gold antibody test was also performed for asymptomatic infected cases in conjunction with nucleic acid testing. Confirmed COVID-19 cases in the laboratories required to meet one of the following two conditions:

1. IgM and IgG antibodies to SARS-CoV-2 are positive;
2. IgG antibody to SARS-CoV-2 is from negative into positive or it has elevated more than 4 times in the convalescence period in comparison to acute phase.

Virus culture

Virus culture was carried out in third-level biological safety laboratories. During the extraction of nucleic acids using virus culturing matter, the addition of lysates or inactivators must be done in the same laboratory and protective conditions as virus cultures. The addition of lysates or inactivators may be performed in comparison to the protective levels of uncultured infectious materials.

Supplementary note 2. Diagnosis and definition of confirmed cases with COVID-19

Note to S2: This is an English summary of key information from National Guidelines for the Prevention and Control of COVID-19 (5th edition, in Chinese)

Since the COVID-19 outbreak occurred in Wuhan in December 2019, the Chinese authorities have thus far released seven editions of the Diagnosis and Treatment Scheme to incorporate the most updated information into the Scheme. The 1st edition was released on January 15, which only classified critical and non-critical cases; the 2nd edition was released on January 18, which further classified cases into mild/moderate, severe and critical cases; the 3rd edition was released on January 22, which slightly modified the criteria for definition of severe case without changes for other aspects; the 4th edition as released on January 27, which further classified mild/moderate cases into mild cases and moderate cases; The 5th edition was released on February 5, the 6th edition on February 19, and the 7th edition on March 3. Since the 4th edition, the definitions of clinical severity remained the same.

In our dataset, a confirmed case was defined based on epidemiological history (including cluster transmission), clinical manifestations (fever and respiratory symptoms; laboratory evidence of normal or decreased number of leukocytes and/or lymphopenia), lung imaging, and results of SARS-CoV-2 nucleic acid detection. In the 7th edition, detection of serum-specific antibodies was further added. The classification of clinical severity of COVID-19 cases is shown below.

Mild case was referred to the clinical symptoms are mild and no pneumonia manifestations can be found in imaging.

Moderate case was defined as patients have symptoms such as fever and respiratory tract symptoms etc., and pneumonia manifestations can be seen in imaging.

Severe case was referred to patients who meet any of the following criteria: dyspnea or respiratory rate ≥30 breaths/min; oxygen saturation ≤93% at a rest state; arterial partial pressure of oxygen (PaO2)/oxygen concentration (FiO2) ≤300 mmHg. Patients with >50% lesions progression within 24 to 48 hours in lung imaging should be treated as severe cases.

Critical case was referred to patients who meet any of the following criteria: occurrence of respiratory failure requiring mechanical ventilation; presence of shock; other organ failure that requires monitoring and treatment in the Intensive Care Unit.

Clinically-diagnosed cases were based on the 7th edition of the Scheme released by the National Health Commission of China released. A presumptive case was defined as meeting the following criteria: (1) close contact with a confirmed or probable case; or cluster transmission; (2) fever and/or respiratory symptoms; (3) laboratory evidence of normal or decreased number of leukocytes and/or lymphopenia; and (4) those presumptive cases with further radiographic evidence showing pneumonia but without a positive RT-PCR test result were defined as clinically-diagnosed cases.

Supplementary Table 1. Median age-stratified Ct value of the samples of asymptomatic cases

Age (years)	Ct value_ ORF	Ct value_ N
	Median (IQR)	Median (IQR)
6-17	34.69(4.98)	34.77(4.95)
18-44	35.63(4.67)	34.47(4.68)
45-64	34.63(5.65)	34.00(4.39)
65+	35.28(5.36)	34.85(4.05)

Note: IQR, interquartile range; ORF, open reading frame; N, nucleocapsid protein
