# Supplementary material for: Post-lockdown SARS-CoV-2 nucleic acid screening in nearly ten million residents of Wuhan, China
Source: Nat Commun. 2020 Nov 20;11:5917. doi: 10.1038/s41467-020-19802-w (PMC7679396; doi:10.1038/s41467-020-19802-w)
Supplement: Supplementary file 2 — Reporting Summary [file 41467_2020_19802_MOESM2_ESM.pdf]

nature research

Corresponding author(s): DBPR -NCOMMS-20-33366A

Last updated by author(s): Oct 21, 2020

Reporting Summary

Nature Research wishes to improve the reproducibility of the work that we publish. This form provides structure for consistency and transparency in reporting. For further information on Nature Research policies, see our Editorial Policies and the Editorial Policy Checklist.

Statistics

For all statistical analyses, confirm that the following items are present in the figure legend, table legend, main text, or Methods section.

n/a	Confirmed	
x		The exact sample size (*n*) for each experimental group/condition, given as a discrete number and unit of measurement
	x	A statement on whether measurements were taken from distinct samples or whether the same sample was measured repeatedly
	x	The statistical test(s) used AND whether they are one- or two-sided<br>*Only common tests should be described solely by name; describe more complex techniques in the Methods section.*
	x	A description of all covariates tested
x		A description of any assumptions or corrections, such as tests of normality and adjustment for multiple comparisons
	x	A full description of the statistical parameters including central tendency (e.g. means) or other basic estimates (e.g. regression coefficient) AND variation (e.g. standard deviation) or associated estimates of uncertainty (e.g. confidence intervals)
	x	For null hypothesis testing, the test statistic (e.g. *F*, *t*, *r*) with confidence intervals, effect sizes, degrees of freedom and *P* value noted<br>*Give P values as exact values whenever suitable.*
x		For Bayesian analysis, information on the choice of priors and Markov chain Monte Carlo settings
x		For hierarchical and complex designs, identification of the appropriate level for tests and full reporting of outcomes
x		Estimates of effect sizes (e.g. Cohen's *d*, Pearson's *r*), indicating how they were calculated

Our web collection on statistics for biologists contains articles on many of the points above.

Software and code

Policy information about availability of computer code

Data collection	Before sample collection, residents electronically (using a specifically designed smartphone application) self-uploaded their personal information, including ID number, name, sex, age, and place of residence. Then, the electronic machine system generated a unique personal barcode and stuck it on the sample tube to ensure the match between the sample and the participant. Then trained staff interviewed each individual regarding the history of COVID-19 and previous nucleic acid testing. There was a database of confirmed COVID-19 cases in Wuhan, which can be used to validate the self-reported previous COVID-19 infection. All information was entered into a central database. The testing results were continually uploaded to the central database by testing institutions. Contact tracing investigations were conducted on participants who tested positive for SARS-CoV-2, to track and manage their close contacts. The pre-existing unique identification code for each resident was used as the programme's identification number, to ensure information accuracy during the whole process of screening, from sampling, nucleic acid testing, result reporting, the isolation of detected positive cases, and tracing of close contacts of positive cases. All screening information was kept strictly confidential and was not allowed to be disclosed or used for other purposes other than clinical and public health management. Personal information of asymptomatic positive cases was only disclosed to designated medical institutions and community health centres for the purpose of medical isolation and identification of close contacts. Researcher was blind to the study hypothesis during data collection.
Data analysis	Detection rate of asymptomatic positive or re-positive cases was calculated by dividing the number of individuals with a positive result of nucleic acid testing by the number of participants tested. Because of extremely low detection rates, we calculated 95% confidence intervals of estimated proportions using Pearson-Klopper exact method, implemented through R package "binom" version 1.1-118. SPSS version 22.0 was used for other statistical analyses. We analyzed the distribution of asymptomatic positive cases and assessed the Spearman correlation between the asymptomatic positive rate and the prevalence of previously confirmed COVID-19 cases in different districts of Wuhan. Differences in asymptomatic positive rates by sex and age groups were assessed using the $\chi 2$ test. ArcGIS 10.0 was used to draw a geographic distribution map of asymptomatic positive cases. A value of P<0.05 (two-tailed) was considered statistically significant.

For manuscripts utilizing custom algorithms or software that are central to the research but not yet described in published literature, software must be made available to editors and reviewers. We strongly encourage code deposition in a community repository (e.g. GitHub). See the Nature Research guidelines for submitting code & software for further information.

Data

Policy information about availability of data

All manuscripts must include a data availability statement. This statement should provide the following information, where applicable:

- Accession codes, unique identifiers, or web links for publicly available datasets
- A list of figures that have associated raw data
- A description of any restrictions on data availability

Detailed data directly used to generate each figure or table of this study are available within the article and Supplements of “Source data”.

Field-specific reporting

Please select the one below that is the best fit for your research. If you are not sure, read the appropriate sections before making your selection.

☐ Life sciences ☒ Behavioural & social sciences ☐ Ecological, evolutionary & environmental sciences

For a reference copy of the document with all sections, see nature.com/documents/nr-reporting-summary-flat.pdf

Behavioural & social sciences study design

All studies must disclose on these points even when the disclosure is negative.

Study description	Quantitative cross-sectional study
Research sample	The screening programme recruited residents (including recovered COVID-19 patients) currently living in Wuhan who were aged ≥6 years
Sampling strategy	All city residents who were aged ≥6 years were recruited to attend the citywide nucleic acid screening.
Data collection	Before sample collection, residents electronically (using a specifically designed smartphone application) self-uploaded their personal information, including ID number, name, sex, age, and place of residence. Then, the electronic machine system generated a unique personal barcode and stuck it on the sample tube to ensure the match between the sample and the participant. Then trained staff interviewed each individual regarding the history of COVID-19 and previous nucleic acid testing. There was a database of confirmed COVID-19 cases in Wuhan, which can be used to validate the self-reported previous COVID-19 infection. All information was entered into a central database. The testing results were continually uploaded to the central database by testing institutions. Contact tracing investigations were conducted on participants who tested positive for SARS-CoV-2, to track and manage their close contacts. The pre-existing unique identification code for each resident was used as the programme’s identification number, to ensure information accuracy during the whole process of screening, from sampling, nucleic acid testing, result reporting, the isolation of detected positive cases, and tracing of close contacts of positive cases. All screening information was kept strictly confidential and was not allowed to be disclosed or used for other purposes other than clinical and public health management. Personal information of asymptomatic positive cases was only disclosed to designated medical institutions and community health centres for the purpose of medical isolation and identification of close contacts. Researcher was blind to the study hypothesis during data collection.
Timing	The city government conducted a citywide nucleic acid screening of SARS-CoV-2 Infection between May 14 and June 1, 2020.
Data exclusions	No data were excluded from the analyses.
Non-participation	No participants declined participation.
Randomization	This study is a census. All samples participated in the citywide nucleic acid screening of SARS-CoV-2 Infection.

Reporting for specific materials, systems and methods

We require information from authors about some types of materials, experimental systems and methods used in many studies. Here, indicate whether each material, system or method listed is relevant to your study. If you are not sure if a list item applies to your research, read the appropriate section before selecting a response.

Materials & experimental systems

n/a	Involved in the study
☒	☐ Antibodies
☒	☐ Eukaryotic cell lines
☒	☐ Palaeontology and archaeology
☒	☐ Animals and other organisms
☐	☒ Human research participants
☒	☐ Clinical data
☒	☐ Dual use research of concern

Methods

n/a	Involved in the study
☒	☐ ChIP-seq
☒	☐ Flow cytometry
☒	☐ MRI-based neuroimaging

Human research participants

Policy information about studies involving human research participants

Population characteristics	There were 10,652,513 eligible people aged ≥6 years in Wuhan (94.1% of the total population). The nucleic acid screening was completed in 19 days (from May 14, 2020 to Jun 1, 2020), and tested a total of 9,899,828 persons from the 10,652,513 eligible people (participation rate, 92.9%). Of the 9899,828 participants, 9865,404 had no previous diagnosis of COVID-19, and 34,424 were recovered COVID-19 patients. No new symptomatic cases and 300 asymptomatic cases (detection rate 0.303/10,000, 95% CI 0.270-0.339/10,000) were identified. There were no positive tests amongst 1,174 close contacts of asymptomatic cases. 107 of 34,424 previously recovered COVID-19 patients tested positive again (re-positive rate 0.31%, 95% CI 0.423-0.574%)
Recruitment	A census was employed in this study, and no selection bias existed . A citywide nucleic acid screening group was formed, with specialized task teams contributing to comprehensive coordination, technical guidance, quality control, participation invitation, information management, communication, and supervision of the screening. The city government invested 900 million yuan (RMB) in the testing programme. From 14 May to 1 June 2020, in the peak time, up to 2907 sample collection sites were functioning at the same time in Wuhan. Each sample collection site had an assigned sample collection group, including several health professionals (staffed according to the number of communities' residents), 2-4 community managers, 1-2 police officers, and 1-2 inspectors. The sampling sites were set up based on the number and accessibility of local residents. Local community workers were responsible for a safe and orderly sampling process to minimise the waiting time. In addition, mobile sampling teams were formed by primary health care professionals and volunteers to conduct door-to-door sampling for residents who had physical difficulties or were unable to walk.
Ethics oversight	All participants provided written or verbal informed consent after reading a statement that explained the purpose of the testing. For participants who aged 6-17 years old, consent was obtained from their parents or guardians. The study protocol for an evaluation of the programme based on anonymized screening data was approved by the Ethics Committee of the Tongji Medical College Institutional Review Board, Huazhong University of Science and Technology, Wuhan, China (No. IROG0003571).

Note that full information on the approval of the study protocol must also be provided in the manuscript.
